# Intravitreal ranibizumab injection is associated with an increased risk of chronic kidney disease: a population-based study in Taiwan

**DOI:** 10.1007/s00210-023-02910-x

**Published:** 2023-12-28

**Authors:** Chang-Hsu Chen, Paik Seong Lim, Tsai-Kun Wu, Wu-Lung Chuang, Teng-Shun Yu, Fuu-Jen Tsai, Chuan-Mu Chen, Kuang-Hsi Chang

**Affiliations:** 1 Ph.D. Program in Translational Medicine, National Chung Hsing University, Taichung, 402 Taiwan; 2 Division of Renal Medicine, Tungs’ Taichung MetroHarbor Hospital, Taichung, 435 Taiwan; 3 Rong Hsing Research Center for Translational Medicine, National Chung Hsing University, Taichung, 402 Taiwan; 4 Department of Life Sciences, College of Life Sciences, National Chung Hsing University, Taichung, 402 Taiwan; 5 Post Baccalaureate Medicine, National Chung Hsing University, Taichung, 402 Taiwan; 6 Division of Endocrinology and Metabolism, Department of Internal Medicine, Changhua Christian Hospital, Changhua, 500 Taiwan; 7 Division of Endocrinology and Metabolism, Department of Internal Medicine, Lukang Christian Hospital, Changhua, 505 Taiwan; 8 Management Office for Health Data, China Medical University Hospital, Taichung, 404 Taiwan; 9 College of Medicine, China Medical University, Taichung, 404 Taiwan; 10 School of Chinese Medicine, College of Chinese Medicine, China Medical University, Taichung, 404 Taiwan; 11 Department of Medical Research, China Medical University Hospital, China Medical University, Taichung, 404 Taiwan; 12 Division of Medical Genetics, China Medical University Children’s Hospital, Taichung, 404 Taiwan; 13 Department of Biotechnology and Bioinformatics, Asia University, Taichung, 413 Taiwan; 14 The iEGG and Animal Biotechnology Center, National Chung Hsing University, Taichung, 402 Taiwan; 15 Department of Medical Research, Tungs’ Taichung MetroHarbor Hospital, Taichung, 435 Taiwan; 16 Center for General Education, China Medical University, Taichung, 404 Taiwan; 17 General Education Center, Nursing and Management, Jen-Teh Junior College of Medicine, Miaoli, 356 Taiwan

**Keywords:** Ranibizumab, Vascular endothelial growth factor (VEGF), Chronic kidney disease, National Health Insurance Research Database (NHIRD)

## Abstract

Systemic vascular endothelial growth factor (VEGF) blockade has been the top adjunctive chemotherapy since 1990. Anti-VEGF therapy has also been associated with worsened renal function in some patients. However, the association between patient outcomes and use of intravitreal VEGF inhibitors remains controversial. Thus, it is necessary to determine the action mechanism and long-term renal effects of ranibizumab. The National Health Insurance Research Database (NHIRD) is one of the largest global databases that are extensively used for epidemiological research. NHIRD contains the medical information of all insureds, such as inpatient, outpatient, emergency, and traditional Chinese medicine records. We selected subjects aged ≥ 20 years who recently administered ranibizumab for the ranibizumab cohort. Non-ranibizumab cohort consisted of subjects who did not receive ranibizumab, and the index date was a random date between 2008 and 2018. We excluded subjects with missing sex and age records and those in which the date of primary outcome was before the index date. The two cohorts were matched via 1:1 propensity score matching based on sex, age, index year, hypertension, diabetes mellitus, hyperlipidemia, stroke, coronary artery disease, alcoholism, chronic obstructive pulmonary disease, and age-related macular degeneration, retinal vein occlusion, and diabetic macular edema. Medical confounders were angiotensin I-converting enzyme inhibitors, statins, corticosteroids, VEGF inhibitors including bevacizumab and aflibercept, lithium, amphotericin B, adefovir, NSAIDS, cisplatin, and calcineurin inhibitors. Among 48,248 participants aged ≥ 20 years, 24,136 (50%) received ranibizumab (13,565 male [56.20%] and 10,571 female [43.80%]). Moreover, 24,136 participants who did not receive ranibizumab were matched by age, sex, comorbidities, and medications. Subjects who received ranibizumab exhibited a significantly higher risk of CKD than those who did not receive ranibizumab (adjusted hazard ratio = 1.88, 95% CI = 1.79–1.96). Our findings revealed that exposure to intravitreal ranibizumab is an independent risk factor for CKD. Therefore, physicians and ophthalmologists should make the patients aware of such a correlation to increase patient safety and decrease the CKD burden.

## Introduction

Systemic vascular endothelial growth factor (VEGF) blockade has been the top adjunctive chemotherapy since 1990 (Hanna et al. 2019). Anti-angiogenic agents are widely used to treat various malignancies, such as non-small cell lung cancer, renal cell carcinoma, and colorectal cancer (Hanna et al. 2019; Phadke et al. 2021). VEGF inhibitors are powerful tools used to delay neovascularization and prevent retinal damage (Phadke et al. 2021). Intravitreal anti-VEGF therapy is widely used for the treatment of proliferative diabetic retinopathy/diabetic macular edema, age-related macular degeneration, and central retinal vein occlusion (Hanna et al. 2019; Hanna et al. 2022). Bevacizumab was the first anti-VEGF antibody used off-label via intravitreal injections to treat retinal neovascularization (Hanna et al. 2022). Aflibercept and ranibizumab have also been approved by the U.S. Food and Drug Administration for ophthalmological use (Hanna et al. 2019, 2022).

Compared to systemic administration, intravitreal use of anti-VEGF agents was initially considered to be a safer choice (Hanna et al. 2019). However, systemic exposure was reported to be more impactful in later studies (Avery et al. 2014, 2017). Bevacizumab, aflibercept, and ranibizumab are immediately circulated in the body (Avery et al. 2014, 2017). Specifically, ranibizumab is rapidly cleared from the bloodstream and exerts the weakest anti-VEGF effects (Avery et al. 2014, 2017). Aflibercept is the most effective in decreasing the plasma-free VEGF levels (Avery et al. 2014, 2017). Moreover, multiple intravitreal injections prolong the period of detectable systemic exposure of the drug (Avery et al. 2014).

Systemic toxicity of intravitreal VEGF blockade is a serious concern. Several studies have reported that intravitreal anti-VEGF therapy is associated with increased hypertension (Rasier et al. 2009), proteinuria (Chung et al. 2020), and thrombotic events (Schmid et al. 2015; Avery and Gordon 2016). Anti-VEGF therapy has also been associated with worsened renal function in some patients (Diabetic Retinopathy Clinical Research N et al. 2007; Jalalonmuhali et al. 2020). Serial retrospective studies have reported that intravitreal bevacizumab increases the risk of mortality in patients with age-related macular degeneration (Hanhart et al. 2017, 2018a, b). However, the association between patient outcomes and use of intravitreal VEGF inhibitors remains controversial, as some studies have reported no significant effects of these inhibitors on hypertension (Risimic et al. 2013; Glassman et al. 2018), proteinuria (Glassman et al. 2018; O’Neill et al. 2019; Bagheri et al. 2018), renal function (O’Neill et al. 2019; Bagheri et al. 2018; Kameda et al. 2018), and mortality (Dalvin et al. 2019) in patients.

Among currently available intravitreal VEGF inhibitors, ranibizumab is less potent and considered a safer option to treat patients with comorbidities (Hanna et al. 2022). Moreover, ranibizumab exhibited the least systemic exposure and decrease in plasma VEGF levels after intravitreal injection compared to bevacizumab and aflibercept (Avery et al. 2014, 2017). Ranibizumab also reduced nephrotoxicity, without any significant effect on glomeruli, in an animal study (Tschulakow et al. 2014). In contrast, several studies have reported that ranibizumab decreases renal function, worsens proteinuria, and causes thrombotic microangiopathy (Phadke et al. 2021; Pelle et al. 2011; Morales et al. 2017). As it is prioritized in the treatment of patients with comorbidities, it is necessary to determine the action mechanism and long-term renal effects of ranibizumab (Hanna et al. 2019; Estrada et al. 2019).

In this nationwide cohort study, we used propensity score matching to determine the risk of chronic kidney disease (CKD) in patients receiving intravitreal ranibizumab injections in Taiwan.

## Methods

### Data source

Since March, 1995, > 99% of the Taiwanese population has been insured via the National Health Insurance (NHI) program of Taiwan. National Health Insurance Research Database (NHIRD) is one of the largest global databases that are extensively used for epidemiological research. NHIRD contains the medical information of all insureds, such as inpatient, outpatient, emergency, and traditional Chinese medicine records. Patient diagnoses were recorded according to the International Classification of Diseases, 9th Revision, Clinical Modification, and International Classification of Diseases, 10th Revision (ICD-9-CM and ICD-10-CM). All analyses were conducted at the China Medical University branch center of the Ministry of Health and Welfare. This study was approved by the Institutional Review Board of China Medical University (CMUH110-REC3-133).

### Subject inclusion and exclusion criteria

We selected subjects aged ≥ 20 years recently administered ranibizumab for the ranibizumab cohort, and the index date was defined as the first date of ranibizumab treatment between 2008 and 2018. Non-ranibizumab cohort consisted of subjects who did not receive ranibizumab, and the index date was a random date between 2008 and 2018. We excluded subjects with missing sex and age records and those in which the date of primary outcome was before the index date. The two cohorts were matched via 1:1 propensity score matching based on sex, age, index year, comorbidities, and medications. In the following section, definitions for comorbidities and medications will be expounded upon.

### Primary outcomes, comorbidities, and medications

The primary endpoint was CKD in this study. We followed up the patients until CKD was diagnosed at the end of 2019 or until they withdrew from the NHI. CKD was defined as ICD-9-CM code 585 and ICD-10-CM code N18. Comorbidities with potential confounding factors were extracted from the database from January 1, 2008 to the index date of the subject. CKD-related comorbidities consisted of hypertension (ICD-9-CM code 401–405 and ICD-10-CM codes I10, I11, I12, I13, I15, and N26.2), diabetes mellitus (ICD-9-CM code 250 and ICD-10-CM code E08-E13), hyperlipidemia (ICD-9-CM code 272 and ICD-10-CM codes E71.30, E75.21, E75.22, E75.24, E75.3, E75.5, E75.6, E77, E78.0, E78.1, E78.2, E78.3, E78.4, E78.5, E78.6, E78.70, E78.79, E78.8, and E78.9), stroke (ICD-9-CM code 430–438 and ICD-10-CM code I60-I69), coronary artery disease (CAD; ICD-9-CM code 410–414 and ICD-10-CM code I20-I25), alcoholism (ICD-9-CM codes 291, 303, 305.0, 571.0–571.3, 790.3, V11.3, and V79.1 and ICD-10-CM codes F10, K70, R78.0, and Z65.8), chronic obstructive pulmonary disease (COPD; ICD-9-CM codes 490–496 and 504–506 and ICD-10-CM codes J40-J47 and J64-J68), and age-related macular degeneration (AMD; ICD-9-CM codes 362.52, ICD-10-CM: H35.32), retinal vein occlusion (RVO; ICD-9-CM: 362.36, ICD-10-CM: H34.83), and diabetic macular edema (DME; ICD-9-CM: 362.01, ICD-10-CM: E11.311). Medical confounders were angiotensin I-converting enzyme inhibitors, statins, corticosteroids, VEGF inhibitors including bevacizumab and aflibercept, lithium, amphotericin B (AmB), adefovir, NSAIDS, cisplatin, and calcineurin inhibitors (CNIs). Medication usage was defined as receiving a prescription after the index date.

### Statistical analyses

Standardized mean difference (SMD) was used to estimate the differences in baseline characteristics between the case and control groups. Density of CKD events per 1000 person-years was calculated for both cohorts during the study period. Cox model was used to compare the risk of CKD between the case and control groups. Model 1 included a crude estimate of the hazard ratio and a 95% confidence interval (CI). Model 2 was adjusted for age, sex, comorbidities, and medications, and it estimated the adjusted HR (AHR). Moreover, Kaplan–Meier analysis and log-rank tests were used to estimate the difference in the cumulative incidence of CKD between the two groups.

## Results

Among 48,272 participants aged ≥ 20 years, 24,136 (50%) received ranibizumab (13,565 men [56.20%] and 10,571 women [43.80%]). Moreover, 24,136 participants who did not receive ranibizumab were matched by age, sex, comorbidities, and medications (Table 1), and the baseline characteristics were found to be well-balanced. Mean (standard deviation) ages of subjects in the case and control groups were 66.23 (12.13) and 66.42 years (12.79), respectively. In the non-ranibizumab and ranibizumab cohorts, the top three comorbidities were hypertension (69.84 vs. 72.65%), diabetes mellitus (61.09 vs. 61.71%), and hyperlipidemia (59.40 vs. 64.36%). Compared with those in the control group, subjects in the case group had similar proportions of comorbidities and medications.

**Table 1:** Characteristics of individuals with and without ranibizumab treatment

Covariates	Control (*n* = 24,136)		Ranibizumab (*n* = 24,136)		*SMD*
	*n*	%	*n*	%	
Age					
20 − 49	2051	8.50	1858	7.70	0.029
50 − 64	8666	35.90	8916	36.94	0.022
≥ 65	13,419	55.60	13,362	55.36	0.005
Mean ± SD	66.42	12.79	66.23	12.13	0.015
Gender					0.003
Women	10,535	43.65	10,571	43.80	
Men	13,601	56.35	13,565	56.20	
Comorbidity					
HT	17,536	72.65	16,857	69.84	0.062
DM	15,618	64.71	14,744	61.09	0.075
HL	15,535	64.36	14,336	59.40	0.102
Stroke	4853	20.11	4344	18.00	0.054
CAD	7287	30.19	6427	26.63	0.079
COPD	7197	29.82	6789	28.13	0.037
AMD	4451	18.44	5228	21.66	0.080
RVO	1176	4.87	1115	4.62	0.012
DME	6826	28.28	6026	24.97	0.075
Medication					
ACEI	3831	15.87	3455	14.31	0.044
Statin	14,282	59.17	13,953	57.81	0.028
Corticosteroid	14,734	61.05	14,154	58.64	0.049
VEGF	837	3.47	749	3.10	0.020
Lithium	129	0.53	27	0.11	0.075
AmB	36	0.15	22	0.09	0.017
Adefovir	18	0.07	6	0.02	0.022
NSAIDS	15,486	64.16	15,162	62.82	0.028
Cisplatin	320	1.33	168	0.70	0.063
CNIs	405	1.68	283	1.17	0.043

*SMD* standardized mean difference, *HT* hypertension, *DM* diabetes mellitus, *HL* hyperlipidemia, *CAD* coronary heart disease, *COPD* chronic obstructive pulmonary disease, *AMD* age-related macular degeneration, *RVO* retinal vein occlusion, *DME* diabetic macular edema, *ACEI* angiotensin-converting enzyme inhibitors, *VEGF* inhibitors including bevacizumab and aflibercept, AmB amphotericin B, *NSAIDS* non-steroidal anti-inflammatory drugs, *CNIs* calcineurin inhibitors

Table 2 presents the association between CKD incidence and patients with and without ranibizumab treatment. Subjects who received ranibizumab exhibited a significantly higher risk of CKD than those who did not receive ranibizumab (AHR = 1.88, 95% CI = 1.79–1.96). Compared with the female subjects, male subjects had higher risk of CKD (AHR = 1.29, 95% CI = 1.23–1.35). Compared with subjects in the 20–49 age group, subjects in the 50–64 age group and those > 65 years had a lower risk of CKD. The adjusted HRs were 0.76 (0.70–0.83) and 0.82 (0.75–0.89), respectively. In addition, patients with hypertension (adjusted HR = 1.86, 95% CI = 1.73–1.99), diabetes mellitus (adjusted HR = 2.37, 95% CI = 2.21–2.54), hyperlipidemia (adjusted HR = 1.19, 95% CI = 1.13–1.26), stroke (adjusted HR = 1.16, 95% CI = 1.10–1.23), CAD (adjusted HR = 1.24, 95% CI = 1.18–1.30), and COPD (adjusted HR = 1.10, 95% CI = 1.04–1.15) exhibited significantly higher risks of CKD than the corresponding groups (AHR > 1; *p* < 0.05). Moreover, patients who received statin, VEGF inhibitor, lithium, and NSAIDS had significantly lower risks of CKD than the corresponding groups (aHR < 1; *p* < 0.05).

**Table 2:** Risk factor analyses of all subjects for chronic kidney disease (CKD)

Covariates		Event	PY	IR	cHR	95% CI	aHR	95% CI
Ranibizumab	No	2921	89,775	32.54	1.00		1.00	
	Yes	4865	89,860	54.14	1.67	1.59, 1.75	1.88	1.79, 1.96
Age	20 − 49	647	13,071	49.50	1.00		1.00	
	50 − 64	2840	66,436	42.75	0.87	0.80, 0.95	0.76	0.70, 0.83
	≥ 65	4299	100,127	42.94	0.88	0.81, 0.96	0.82	0.75, 0.89
Gender	Women	2967	78,381	37.85	1.00		1.00	
	Men	4819	101,254	47.59	1.26	1.21, 1.32	1.29	1.23, 1.35
Comorbidity								
HT	No	1162	55,400	20.97	1.00		1.00	
	Yes	6624	124,234	53.32	2.52	2.37, 2.68	1.86	1.73, 1.99
DM	No	1320	72,052	18.32	1.00		1.00	
	Yes	6466	107,583	60.10	3.25	3.06, 3.45	2.37	2.21, 2.54
HL	No	2256	72,107	31.29	1.00		1.00	
	Yes	5530	107,528	51.43	1.63	1.55, 1.71	1.19	1.13, 1.26
Stroke	No	5991	147,641	40.58	1.00		1.00	
	Yes	1795	31,994	56.11	1.38	1.30, 1.45	1.16	1.1, 1.23
CAD	No	5226	130,102	40.17	1.00		1.00	
	Yes	2560	49,533	51.68	1.29	1.23, 1.35	1.24	1.18, 1.30
Alcoholism	No	7636	176,816	43.19	1.00		1.00	
	Yes	150	2819	53.22	1.22	1.04, 1.43	1.23	1.04, 1.44
COPD	No	5512	129,320	42.62	1.00		1.00	
	Yes	2274	50,315	45.20	1.06	1.01, 1.11	1.1	1.04, 1.15
AMD	No	6679	140,333	47.59	1.00		1.00	
	Yes	1107	39,301	28.17	0.6	0.56, 0.64	0.9	0.84, 0.97
RVO	No	7537	173,260	43.50	1.00		1.00	
	Yes	249	6374	39.06	0.88	0.78, 1.00	0.83	0.73, 0.94
DME	No	4553	133,506	34.10	1.00		1.00	
	Yes	3233	46,128	70.09	2.04	1.95, 2.13	1.36	1.30, 1.43
Medication								
ACEI	No	6372	149,160	42.72	1.00		1.00	
	Yes	1414	30,474	46.40	1.10	1.04, 1.16	0.99	0.94, 1.05
Statin	No	2578	69,858	36.90	1.00		1.00	
	Yes	5208	109,777	47.44	1.29	1.23, 1.35	0.75	0.71, 0.80
Corticosteroid	No	2099	68,971	30.43	1.00		1.00	
	Yes	5687	110,664	51.39	1.69	1.61, 1.78	1.1	1.04, 1.16
VEGF	No	7649	172,996	44.21	1.00		1.00	
	Yes	137	6639	20.64	0.47	0.40, 0.56	0.55	0.46, 0.65
Lithium	No	7779	178,945	43.47	1.00		1.00	
	Yes	7	690	10.15	0.24	0.11, 0.50	0.31	0.15, 0.66
AmB	No	7776	179,401	43.34	1.00		1.00	
	Yes	10	234	42.81	0.99	0.53, 1.84	1.4	0.75, 2.61
Adefovir	No	7781	179,527	43.34	1.00		1.00	
	Yes	5	107	46.59	1.10	0.46, 2.64	1.59	0.66, 3.81
NSAIDS	No	3935	51,495	76.42	1.00		1.00	
	Yes	3851	128,140	30.05	0.40	0.38, 0.41	0.41	0.39, 0.43
Cisplatin	No	7743	178,014	43.50	1.00		1.00	
	Yes	43	1621	26.53	0.61	0.45, 0.82	0.83	0.61, 1.12
CNIs	No	7677	176,770	43.43	1.00		1.00	
	Yes	109	2865	38.05	0.89	0.73, 1.07	1.13	0.93, 1.37

*Event* number of patients with of CKD, *PY* person-year, IR incidence rate (per 1000 person-years), *cHR* crude hazard ratio, *aHR* adjusted hazard ratio adjusted for age, gender, comorbidities, and medications, *CI* confidence interval, *HT* hypertension, *DM* diabetes mellitus, *HL* hyperlipidemia, *CAD* coronary heart disease, *COPD* chronic obstructive pulmonary disease, *AMD* age-related macular degeneration, *RVO* retinal vein occlusion, *DME* diabetic macular edema, *ACEI* angiotensin converting enzyme inhibitors, *VEGF* inhibitors including bevacizumab and aflibercept, *AmB* amphotericin B, *NSAIDS* non-steroidal anti-inflammatory drugs, *CNIs* calcineurin inhibitors

Table 3 presents the subgroup analysis results: subjects with varying sex, age, comorbidities, and medications; ranibizumab users tended to have a lower risk of CKD than non-ranibizumab users (adjusted HR > 1, *p*-value < 0.05).

**Table 3:** Incidence and hazard ratio of CKD in individuals with and without ranibizumab treatment based on age, gender, comorbidities, and medications

Covariates		Control			Ranibizumab			cHR	(95% CI)	aHR	(95% CI)
		Event	PY	IR	Event	PY	Rate				
Age	20 − 49	157	7808	20.11	490	5263	93.10	4.50	3.76, 5.40	4.50	3.76, 5.40
	50 − 64	906	33,992	26.65	1934	32,443	59.61	2.22	2.06, 2.41	2.22	2.06, 2.41
	≥ 65	1858	47,974	38.73	2441	52,154	46.80	1.21	1.14, 1.29	1.21	1.14, 1.29
Gender	Women	1129	39,410	28.65	1838	38,971	47.16	1.65	1.53, 1.77	1.64	1.52, 1.77
	Men	1792	50,365	35.58	3027	50,889	59.48	1.68	1.59, 1.78	1.69	1.60, 1.80
Comorbidity											
HT	No	386	26,183	14.74	776	29,217	26.56	1.80	1.59, 2.03	1.81	1.60, 2.05
	Yes	2535	63,592	39.86	4089	60,643	67.43	1.7	1.61, 1.78	1.72	1.63, 1.81
DM	No	581	32,800	17.71	739	39,252	18.83	1.05	0.94, 1.17	0.99	0.89, 1.10
	Yes	2340	56,975	41.07	4126	50,608	81.53	1.98	1.88, 2.08	1.98	1.89, 2.09
HL	No	839	32,806	25.57	1417	39,301	36.06	1.41	1.30, 1.54	1.40	1.29, 1.53
	Yes	2082	56,968	36.55	3448	50,559	68.20	1.87	1.77, 1.97	1.87	1.78, 1.98
Stroke	No	2178	73,099	29.80	3813	74,542	51.15	1.72	1.63, 1.81	1.73	1.64, 1.82
	Yes	743	16,676	44.56	1052	15,318	68.68	1.54	1.41, 1.70	1.56	1.42, 1.71
CAD	No	1840	63,824	28.83	3386	66,277	51.09	1.78	1.68, 1.88	1.79	1.69, 1.89
	Yes	1081	25,950	41.66	1479	23,583	62.72	1.51	1.39, 1.63	1.50	1.39, 1.63
Alcoholism	No	2846	87,964	32.35	4790	88,852	53.91	1.67	1.59, 1.75	1.67	1.60, 1.75
	Yes	75	1811	41.42	75	1008	74.42	1.82	1.32, 2.51	1.90	1.37, 2.62
COPD	No	1980	64,595	30.65	3532	64,725	54.57	1.78	1.69, 1.88	1.79	1.70, 1.89
	Yes	941	25,180	37.37	1333	25,135	53.03	1.42	1.31, 1.55	1.41	1.30, 1.53
AMD	No	2442	73,854	33.07	4237	66,479	63.73	1.92	1.83, 2.02	1.92	1.83, 2.02
	Yes	479	15,921	30.09	628	23,381	26.86	0.88	0.78, 1.00	0.90	0.80, 1.02
RVO	No	2775	86,137	32.22	4762	87,123	54.66	1.70	1.62, 1.78	1.71	1.63, 1.79
	Yes	146	3637	40.14	103	2737	37.63	0.94	0.73, 1.21	0.94	0.73, 1.21
DME	No	1592	64,629	24.63	2961	68,878	42.99	1.75	1.65, 1.86	1.74	1.64, 1.85
	Yes	1329	25,146	52.85	1904	20,982	90.74	1.70	1.59, 1.83	1.70	1.59, 1.83
Medication											
ACEI	No	2353	74,002	31.80	4019	75,158	53.47	1.69	1.60, 1.78	1.69	1.60, 1.77
	Yes	568	15,772	36.01	846	14,702	57.54	1.60	1.44, 1.78	1.63	1.47, 1.82
Statin	No	991	33,432	29.64	1587	36,425	43.57	1.49	1.37, 1.61	1.47	1.36, 1.59
	Yes	1930	56,342	34.25	3278	53,435	61.35	1.79	1.70, 1.90	1.81	1.71, 1.91
Corticosteroid	No	792	32,613	24.28	1307	36,358	35.95	1.50	1.37, 1.64	1.48	1.35, 1.62
	Yes	2129	57,161	37.25	3558	53,502	66.50	1.79	1.70, 1.89	1.81	1.72, 1.91
VEGF	No	2857	86,566	33.00	4792	86,430	55.44	1.68	1.61, 1.76	1.69	1.61, 1.77
	Yes	64	3209	19.95	73	3430	21.28	1.03	0.74, 1.45	1.04	0.74, 1.46
NSAIDS	No	1379	25,862	53.32	2556	25,633	99.72	1.87	1.75, 1.99	1.87	1.75, 1.99
	Yes	1542	63,912	24.13	2309	64,227	35.95	1.48	1.39, 1.58	1.47	1.38, 1.57
Cisplatin	No	2899	88,766	32.66	4844	89,248	54.28	1.67	1.59, 1.74	1.67	1.59, 1.75
	Yes	22	1009	21.80	21	612	34.31	1.44	0.79, 2.63	1.34	0.72, 2.50
CNIs	No	2866	88,023	32.56	4811	88,747	54.21	1.67	1.60, 1.75	1.67	1.60, 1.75
	Yes	55	1752	31.40	54	1113	48.51	1.59	1.09, 2.31	1.52	1.03, 2.22

*Event* number of patients with of CKD, *PY* person-year, *IR* incidence rate (per 1000 person-years), *cHR* crude hazard ratio, *aHR* adjusted hazard ratio, adjusted for age, gender, comorbidities, and medications, *CI* confidence interval, *HT* Hypertension, *DM* diabetes mellitus, *HL* hyperlipidemia, *CAD* coronary heart disease, *COPD* chronic obstructive pulmonary disease, *AMD* age-related macular degeneration, *RVO* retinal vein occlusion, *DME* diabetic macular edema, *ACEI* angiotensin converting enzyme inhibitors, *VEGF* inhibitors including bevacizumab and aflibercept, *AmB* amphotericin B, *NSAIDS* non-steroidal anti-inflammatory drugs, *CNIs* calcineurin inhibitors

**Fig. 1:**
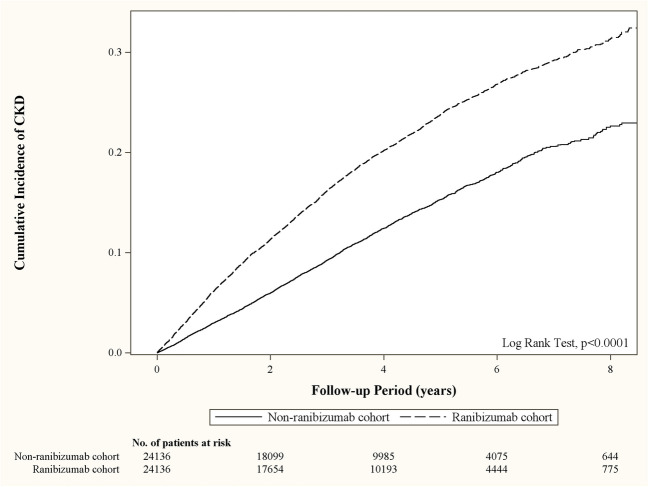
Comparison of cumulative incidence of CKD between patients receiving ranibizumab and those not receiving the treatment

As shown in Fig. 1, the cumulative incidence of CKD in the ranibizumab cohort was significantly higher than that in the non-ranibizumab cohort (log-rank test *p* < 0.001).

## Discussion

Our findings provide evidence that intravitreal ranibizumab, a VEGF inhibitor, is associated with a higher risk of CKD. Using propensity score matching, we created a control group with a comparable distribution to the ranibizumab group regarding gender, age, comorbidities, and medication. Multivariate analysis revealed increased risk of CKD in the ranibizumab group. Subgroup analysis also revealed that ranibizumab was associated with a higher risk of CKD.

To the best of our knowledge, this is the first population-based study to highlight the correlation between intravitreal anti-VEGF therapy and long-term renal patient outcomes. Although several case studies suggested the nephrotoxicity of intravitreal VEGF inhibitors, only a few focused on their chronic effects on renal function (Hanna et al. 2019, 2022). In a retrospective cohort of 85 diabetic macular edema cases, O’Neill et al. reported no association between glomerular filtration rate decline and intravitreal injections of VEGF inhibitors, mainly ranibizumab, in a mean duration of 31 months (O’Neill et al. 2019). Another randomized study of 660 participants with diabetic macular edema revealed that urine albumin–creatinine ratio does not change significantly after intravitreal aflibercept, bevacizumab, and ranibizumab treatment for 52 weeks (Glassman et al. 2018). The first study cohort may be too small to detect rare renal toxicities (O’Neill et al. 2019). In the second study cohort, chronic renal outcomes may not be affected due to the short following period (Glassman et al. 2018). Most studies only focused on diabetic patients with macular edema. Therefore, our study provides robust evidence for the association between intravitreal use of ranibizumab and increased risk of CKD over a long period using a nationwide database. Meanwhile, ranibizumab was selected as the representative treatment in our study because intravitreal bevacizumab is non-reimbursable, and its data is lacking in NHID. Ranibizumab was the first intravitreal anti-VEGF therapy reimbursed by NHI, and it was more clinically experienced than aflibercept before 2018. Although some aflibercept users might be included in the control group, the risk of ranibizumab was still significant. Since our study showed that even less potent and safer ranibizumab was associated with an increased risk of CKD, it is worthwhile to include aflibercept to compare their effects on kidney function and investigate whether drug class effects of adverse long-term renal outcomes exist by using an updated database in the future study.

In Taiwan, old age is associated with a high risk of CKD (Kuo et al. 2007), and CKD is more prevalent in group aged > 65 years (Wen et al. 2008). Age distribution of our cohort revealed that the patients treated with ranibizumab who were eligible for reimbursement, such as those with diabetic macular edema, age-related macular degeneration, polypoidal choroidal vasculopathy, and central or branch retinal vein occlusion with macular edema, were mainly elderly patients. As the elderly are more susceptible to renal injury, several measures have been proposed to minimize the nephrotoxicity of VEGF inhibitors (Estrada et al. 2019). Interestingly, our study revealed a higher risk of developing CKD in the group aged < 50 years after adjusting several covariates including diabetes. Here, we inferred that the young ranibizumab users mostly consisted of patients with diabetic macular edema based on previous epidemiological reports (Ho et al. 2008; Chang et al. 2018; Chang and Wu 2009). Since the development of diabetic retinopathy is correlated with the duration of diabetes (Zhang et al. 2010; Lee et al. 2015), the age of diabetic onset in young diabetic patients using ranibizumab may be considered earlier than old diabetic users. Several studies have shown that the young age of diabetes onset might be an important risk factor for renal complications (Magliano et al. 2020; Wu et al. 2021; Lee et al. 2023). One recent large prospective cohort study in China revealed that the young age of onset of diabetes synergistically enhanced the risk of CKD among the influence of diabetes duration (Wu et al. 2021). Another large population study in Korea also showed that patients with young-onset diabetes (age < 40 years) had 70% higher risk of developing CKD than the late-onset group (Lee et al. 2023). Our finding was consistent with the previous literatures. Current evidence proposed that more rapid β cell failure and obesity in young-onset patients than in late-onset patients leads to worse disease progression (Magliano et al. 2020). Besides, the correlation between intravitreal VEGF antagonist-related renal damage and diabetic nephropathy has also been reported (Morales et al. 2017; Hanna et al. 2020; Shye et al. 2020). Because our cohort was lacking in several parameters such as oral glucose tolerance test, albuminuria, or body mass index, we were unable to determine the possible mechanisms of higher risk of CKD in young ranibizumab users. Nonetheless, our result highlighted the need for clinical awareness, aggressive treatment, and closer collaboration between nephrologists and ophthalmologists to reduce renal progression in ranibizumab users aged < 50 years.

Mechanisms underlying the associations reported here remain unknown. Anti-VEGF nephrotoxicity is associated with various renal pathological manifestations, such as focal segmental glomerulosclerosis, minimal changes disease, membranous nephropathy, acute interstitial nephritis, and thrombotic microangiopathy (Hanna et al. 2019, 2022; Estrada et al. 2019). Downstream effects of VEGF inhibition on podocytes and glomerular endothelial cells have been proposed (Hanna et al. 2022; Estrada et al. 2019). For example, sequestration of VEGF leads to complement activation and increased nuclear factor-κB signaling in both podocytes and endothelial cells, subsequently causing thrombotic microangiopathy (Estrada et al. 2019). Because patients receiving intravitreal ranibizumab are not required specific monitoring, delayed recognition of subclinical renal injury may potentially occur. Acute kidney injury and CKD are intercorrelated (Okusa et al. 2009; Coca et al. 2012). Notably, patients recovering from acute kidney injury may be at risk of CKD due to nephron loss, incomplete repair, inflammation, fibrosis, and epigenetic changes (Wang and Zhang 2022; Tanemoto et al. 2022). As our study lacked serial clinical and laboratory parameters, such as blood pressure or urine protein levels, further prospective studies are needed to examine the causality and time course of the association between intravitreal VEGF inhibitors and risk of CKD.

This study has several limitations. Although we used the propensity score matching method and adjusted for extensively available covariates, we could not account for other residual confounding factors that contributed to the development of CKD. For example, data on cigarette smoking and obesity, two important risk factors for age-related macular degeneration and CKD (Wen et al. 2008; Chakravarthy et al. 2010), were lacking in our cohort. In addition, control group was selected according to the propensity score, and hence, did not reflect the actual health situation of the general population. This may have resulted in an underestimation of CKD risk. Furthermore, our findings may have surveillance bias as patients receiving ranibizumab were more likely to undergo laboratory examinations that detected CKD due to more frequent contact with the medical care system than those who did not receive ranibizumab. However, Taiwan NHI program enrolled > 99.99% of residents, removed some barriers, and provided free health care in the low urbanization areas (Cheng and Chiang 1997; Huang et al. 2006). Therefore, surveillance bias may be limited.

Using ICD codes to define comorbidities and incident CKD may have decreased the sensitivity of diagnosis and resulted in inaccurate administrative data. Therefore, a sampling bias may have occurred. In addition, we could not analyze whether dose accumulation is associated with CKD as reducing the dose and frequency of intravitreal VEGF inhibitors is necessary for treating high-risk patients (Hanna et al. 2019). Finally, we could not determine whether any distinct disease in ranibizumab users increased the risk of CKD.

The main strength of this study is the use of a large community-based cohort with a 10-year follow-up period. We examined the relationship between drug exposure and long-term patient outcomes in a real-world setting. The robustness of our findings was supported by consistent results after adjusting for various medications and comorbidities. Our study is the first to provide evidence for the adverse effects of intravitreal VEGF inhibitors, including low-potency ranibizumab, and their association with the incidence of CKD (Phadke et al. 2021). As it has the highest incidence and prevalence of end-stage renal disease worldwide (Wen et al. 2008), this association should be further validated to aid the public health in Taiwan.

In summary, our findings revealed that exposure to intravitreal ranibizumab is an independent risk factor for CKD. Therefore, physicians and ophthalmologists should make the patients aware of such a correlation to increase patient safety and decrease the CKD burden. However, the specific causal relationships and underlying mechanisms require further investigation.

## Data Availability

Data are available from the NHIRD published by Taiwan National Health Insurance Bureau. Due to the ‘Personal Information Protection Act’, data cannot be made publicly available (http://nhird.nhri.org.tw/en/index.html).
